# Ranking’s Half Yearly Abstract of the Medical Sciences for July

**Published:** 1852-01

**Authors:** 


ARTICLE III.

*Ranking's Half Yearly Abstract of the Medical Sciences for July,* 1851. (From the American Publishers through S. C. Griggs & Co., Chicago.)

This work which we have had occasion before to notice as one of the best medical periodicals of the day, fully sustains its high reputation in the present number.

It is re-published in this country by Lindsay & Blakiston, Philadelphia, at the low price of one dollar and fifty cents per annum; or to mail subscribers with the postage pre-paid at two dollars.

